# Regulation of Immune Homeostasis via Muramyl Peptides-Low Molecular Weight Bioregulators of Bacterial Origin

**DOI:** 10.3390/microorganisms10081526

**Published:** 2022-07-28

**Authors:** Svetlana V. Guryanova

**Affiliations:** Medical Institute, Peoples’ Friendship University of Russia (RUDN University) of the Ministry of Science and Higher Education of the Russian Federation, 117198 Moscow, Russia; svgur@mail.ru

**Keywords:** innate immunity, NOD2, muramyl peptide, MDP, glucoseeaminylmuramyldipeptide, GMDP, lipopolysaccharide, LPS, inflammation, tolerance

## Abstract

Metabolites and fragments of bacterial cells play an important role in the formation of immune homeostasis. Formed in the course of evolution, symbiotic relationships between microorganisms and a macroorganism are manifested, in particular, in the regulation of numerous physiological functions of the human body by the innate immunity receptors. Low molecular weight bioregulators of bacterial origin have recently attracted more and more attention as drugs in the prevention and composition of complex therapy for a wide range of diseases of bacterial and viral etiology. Signaling networks show cascades of causal relationships of deterministic phenomena that support the homeostasis of multicellular organisms at different levels. To create networks, data from numerous biomedical and clinical research databases were used to prepare expert systems for use in pharmacological and biomedical research with an emphasis on muramyl dipeptides. Muramyl peptides are the fragments of the cell wall of Gram-positive and Gram-negative bacteria. Binding of muramyl peptides with intracellular NOD2 receptors is crucial for an immune response on pathogens. Depending on the microenvironment and duration of action, muramyl peptides possess positive or negative regulation of inflammation. Other factors, such as genetic, pollutions, method of application and stress also contribute and should be taken into account. A system biology approach should be used in order to systemize all experimental data for rigorous analysis, with the aim of understanding intrinsic pathways of homeostasis, in order to define precise medicine therapy and drug design.

## 1. Introduction

Microorganisms that inhabit human skin and mucous membranes take an active part in the formation of the human immune system from the moment of birth. Evolutionarily formed relationships between microorganisms and a macroorganism provide the possibility of sustainable development of the human body in the modern world, which has more than a million lines of bacteria [1]. At the same time, microorganisms that do not have a harmful effect on the human body are commensal. Commensal organisms that inhabit the skin and mucous membranes prevent the colonization of harmful bacteria, they produce substances and vitamins that are vital for humans, and when degraded, they are a source of molecules that keep the human immune system active, providing an adequate response to pathogens not only of a bacterial nature, but also on viruses, fungi and protozoa [2]. In view of the emergence of new microorganisms that cause epidemics in the modern world, increasing nonspecific resistance becomes an urgent task. It is of interest to study low molecular weight compounds of bacterial origin due to their ability to pass through the epidermis and mucous membranes, and thus have a local and systemic effect on the macroorganism, helping to combat pathogens. Low molecular weight bioregulators of bacterial origin include short chain fatty acids, bile acids, fragments of cell walls—lipopolysaccharide and muramyl peptides [2]. Muramyl peptides (MPs) are part of the peptidoglycan that forms the backbone of the cell walls of bacteria—both Gram-positive and Gram-negative. MPs are formed during the degradation of bacteria and are pathogen-associated molecular patterns (PAMPs) that are recognized by innate immune receptors. For decades, MPs have attracted the attention of researchers as promising drugs and vaccine components for combating infectious and oncological diseases due to their ability to affect innate and acquired immunity [3,4,5]. This review is devoted to the analysis of the effect of muramyl peptides on the regulation of immune homeostasis (1) at the level of microorganisms inhabiting the skin and mucous membranes; (2) at the level of cell populations and (3) at the level of intracellular processes. In particular, we highlight progress made toward understanding the positive and negative regulation of inflammation by muramyl peptides.

## 2. Sources of Muramyl Peptides in the Human’s Body

**Figure 1:**
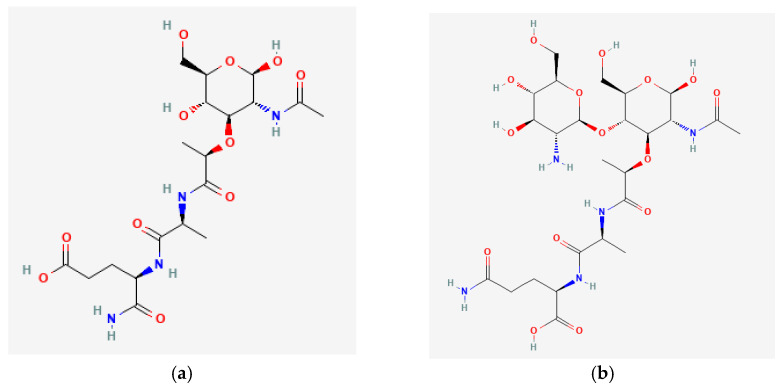
(**a**) N-Acetylmuramyl-L-Alanyl-D-Isoglutamine (MDP); (**b**) N-Acetyl-D-Glucosaminyl-(beta1,4)-N Acetylmuramyl-L-Alanyl-D-Isoglutamine (GMDP).

Muramyl peptides are monomers of peptidoglycan, which forms the cell wall of almost all known bacteria, with the exception of *Rickettsia*, and protect bacteria from osmotic lysis. Gram-positive bacteria have a thicker peptidoglycan layer than Gram-negative bacteria. N-acetylmuramic acid, which is part of muramyl peptides, is a highly conserved structure, synthesized exclusively in prokaryotic organisms, and which, together with N-acetylglucosamine, forms the backbone of bacterial walls. Monosaccharide-containing muramyl peptides (N-acetylmuramyl-L-alanyl-D-isoglutamine, MDP) and disaccharide-containing glucosaminylmuramyl dipeptides (N-acetylglucosaminyl-N-acetylmuramyl-L-alanyl-D-isoglutamine GMDP) (Figure 1) can have modifications as a sugar residue and the peptide part of the molecule, which differ in different bacteria.

The presence or absence of muramyl peptides (MPs) in the organism of a healthy person was a long time of discussion. Methods of mass chromatography (MC), gas liquid chromatography (GLC), and mass spectrometry (MS) were used to determine MPs in human tissues and fluids in order to identify the relationship with chronic infectious foci [6,7,8].

At present, using monoclonal antibodies, it has been found that muramyl peptides are normally present in the blood serum of healthy people at a concentration of 0.330–0.838 μg/mL, where they enter through the intestinal mucosa during the breakdown of microflora [9]. The main role in the degradation of polymeric peptidoglycan is played by host enzymes that cleave bacterial peptidoglycans, for example, lysozyme, amidases, peptidoglycan-recognizing proteins, and many others [10,11]. However, in addition this, bacterial enzymes are also known that use hydrolases, peptidases, amidases, glycosidases, and lysozyme-like proteins for the partial destructuring of cell wall peptidoglycan during bacterial division and growth, pili formation, autolysis, and transmembrane transport [12,13,14]. Enzymes that destroy peptidoglycan are also secreted by bacteria in the competition between bacteria of different lines for the development of new habitat niches and are strictly species-specific [15,16,17]. Bacteriophages also use murein hydrolases and lysozyme-like proteins to penetrate bacteria, destroying their peptidoglycan [18,19,20]. Lysozyme-like proteins that degrade peptidoglycan to disaccharide-containing derivatives have also been found in fungi [21]. Thus, the enzymes of bacteria, bacteriophages, and fungi that are part of the commensal microflora, along with human enzymes, contribute to the formation of muramyl peptides. Recent studies have shown that it is muramyl peptides, due to the production of the cytokine GM-CSF activate tolerogenic CD103+ dendritic cells and provide tolerance to the commensal microflora, maintaining immune homeostasis [22].

In animals, the excretion and degradation of muramyl peptides can occur much faster than in humans. In particular, a study of the content of natural muramyl peptides in the blood serum of rabbits showed that normally muramyl peptides are not detected at a method sensitivity of 500 pmol/mL [23]. Moreover, immediately after the injection of synthetic muramyl dipeptide, it was easily detected in the bloodstream, but this level rapidly decreased [23]. Similarly, in rat tissues with a sensitivity of 1 ng, muramyl peptides were not normally detected, but were determined in the spleen and cerebrospinal fluid of patients with pneumococcal meningitis (from 6.8 to 3900 ng of muramic acid/mL of cerebrospinal fluid) [24]. In mice, after the intravenous and subcutaneous injection of radioactively labeled MDP, more than 50% of 14C-MDP was excreted in the urine after 30 min and more than 90% 2 h after administration; the labeled compound was detected in the urine unchanged [25].

When studying the content of muramyl peptides in the blood serum of laboratory mice using HPLC and MS, the content of various derivatives of muramyl peptides was found [26]. In addition, muramyl peptides GlcNAc-MurNAc-l-Ala-d-Gln-meso DAP (GM-TriDAP), GlcNAc-MurNAc-l-Ala-d-Gln (GMDP) and l-Ala-d-Gln-meso DAP (TriDAP) were found, which suggests that traces of circulating peptidoglycan may be present in serum, and the effect of muramyl peptides in experimental in vitro studies may be underestimated [26].

The species specificity of the ability to cleave muramyl peptides, as well as the presence of muramyl peptides in the serum used for cell culture, must be taken into account when organizing preclinical studies of drugs based on muramyl peptides, as well as when interpreting experimental data from laboratory studies.

## 3. Effect of Muramyl Peptides on Microorganisms

Muramyl peptides, being monomers of peptidoglycan, have a direct effect on microorganisms and their development cycles. Remodeling of peptidoglycan is necessary for bacteria to reproduce; with each division of bacteria, about 40–50% of peptidoglycan breaks down into monomeric units, and can be used to build the cell walls of the next generation of bacteria [27]. When murein hydrolases act on the bacterial cell wall, the muramyl peptides formed in the periplasm are recycled and reused by bacteria to form peptidoglycan [28]. Dozens of murein hydrolases have been described that are characteristic of certain lines of bacteria and bacteriophages, resulting in the formation of a large number of monosaccharide-containing and disaccharide-containing muramyl peptide derivatives that are recycled during bacterial growth [20,29]. Numerous experiments have shown the spontaneous release of peptidoglycan fragments from *E. coli* [30], *Bordetella pertussis* [31,32], *Neisseria gonorrhoeae* [29,32], *Neisseria meningitides* [33], *Bordetella pertussis* [34], and *Shigella* [35].

The direct effect of muramyl peptides and their analogues on five bacterial strains of four species (*S. aureus* MRSA/MSSA, *K. pneumoniae ESBL*, *P. aeruginosa* and *E. coli*) revealed that the value of the minimum inhibitory concentration (MIC) was >512 μg/mL against all strains except for *Pseudomonas aeruginosa (P. aeruginosa)*, for which the MIC was 128 μg/mL. As a comparison, the MICs of antibiotics (kanamycin, tetracycline, and chloramphenicol) in this experiment ranged from 1 to 32 μg/mL [36]. Muramyl peptides can be used by bacteria for communication [37], participating in the coordination of the growth of the entire population and acting, along with other low molecular weight bioregulators of bacterial origin, as a quorum-sensing mediator.

The direct effect of muramyl peptides is also manifested on dormant forms of bacteria: muramyl peptide 1,6-anhydro-GMDP, added to the medium with dormant *Mycobacterium smegmatis* bacteria, revived dormant mycobacterial cells in the concentration range of 9–100 ng/mL [38]. The effect of muramyl peptides on the composition of the microbiocenosis of the oral cavity of healthy patients and patients after caries therapy was shown [39,40]. Oral administration of 1 mg GMDP per day for 10 days resulted in a decrease in oral fluid of *Candida albicans*, *Clostridium difficile*, and *Porphyromonas gingivalis* [39,40].

In experiments on J774 cell lines, GMDP inhibited the intracellular growth of *M. smegmatis*, *M. bovis*, and *M. tuberculosis* at concentrations of 40 μg/mL [41].

The antibacterial activity of a few dozens of muramyl peptide derivatives has been studied in experimental animal models. N-acetylmuramyl-L-alanyl-D-isoglutamine and some analogues have been found to protect mice against *Klebsiella pneumoniae* even when administered orally, and they also proved to be effective when administered after challenge [42,43]. Interesting data have been obtained from intermittent injections of GMDP into mice infected with *Mycobacterium tuberculosis*. It was found that injections of GMDP reduced the number of viable bacilli in the lungs, but increased their number in the spleen after 16 weeks, and also reduced the recurrence of infection in the lungs after chemotherapy [44].

The use of the disaccharide-containing muramyl peptide GMDP 1–4 days before the administration of a lethal dose of *E. coli* LPS endotoxin protects from 60 to 100% of mice from death [45], while the synthetic derivative of MDP protects against *Staphylococcus aureus* [46]. The muramyl dipeptide analogue provided protection against intraperitoneal *Pseudomonas aeruginosa* infection or intravenous *Candida albicans* infection in mice when administered intraperitoneally, intravenously, or subcutaneously at 80 mg/kg once a day for 4 consecutive days prior to infection. The compounds were not active when administered orally. N-acetyl-nor-muramyl-L-alanyl-D-isoglutamine potentiated the action of gentamicin and had a protective effect against *Listeria monocytogenes*. Post-infection protection was not observed [47]. Muramyl peptide MDP-Lys (L18) had a protective effect in C57BL/6 mice against live cultures of *Salmonella enteritidis* and *Salmonella enterica Subspecies enterica* Serovar *Choleraesuis Hokkaido* [48].

Muramyl peptides were able to protect animals with immunodeficiencies from infection [49]. MDP protected CBA/N mice carrying the X-linked immune deficiency mutation (xid) against the lethal bacterial infection *Streptococcus pneumoniae*, *Salmonella typhimurium* and *Salmonella enteritidis* [50]. Interestingly, in another study in a model of *Salmonella enteritidis* in CBA/N-defective mice with X-linked immunodeficiency, a single injection of either MDP or MDP-Lys(L18) did not elicit any effective protection, but repeated injections prior to bacterial challenge of MDP-Lys(L18) (100 µg per mouse per day for 3 consecutive days) protected immunodeficient mice. Multiple injections of MDP once a day for several days in a row significantly increased bactericidal activity in the abdominal cavity and spleen of mice [51]. Muramyl peptides in experimental studies on animals not only demonstrated activity against various bacterial infections, but they also had a protective effect against viruses, fungi, and protozoa [52,53,54].

## 4. Muramyl Peptides Activation of Various Cell Populations

To date, several drugs based on muramyl peptides have been registered, and clinical trials of vaccines containing muramyl peptides are underway [4]. These clinical trials were preceded by numerous studies on the effect of muramyl peptides on various cell populations, which established their ability to activate anti-infective and anti-tumor defenses. With the discovery in 2003 by two independent groups of scientists of the sensors of muramyl peptides—NOD1 and NOD2 proteins and their predominant localization in monocytic cells, the preferential effect of muramyl peptides on the cells of the immune system became clear [55,56]. NOD1 and NOD2 are intracellular receptors of innate immunity; they provide an adequate response to a pathogen in case of its invasion and tolerance to commensal microflora. NOD1 recognizes muramyl peptides containing γ-d-glutamyl-meso-diaminopimelic acid (iE-DAP), a peptdoglycan fragment of Gram-negative bacteria [57]. NOD2 recognizes muramyl peptides containing L-Ala-D-isoGln and its derivatives [55,56]. Mutations in NOD1 and NOD2 are associated with inflammatory [58,59,60], oncological [61], and neurodegenerative diseases [62,63]. NOD1 and NOD2 receptors in monocytes initiate an adequate response and clearance from the pathogen. Derivatives of monocytes are macrophages, dendritic cells, osteoclasts, microglia.

**Figure 2:**
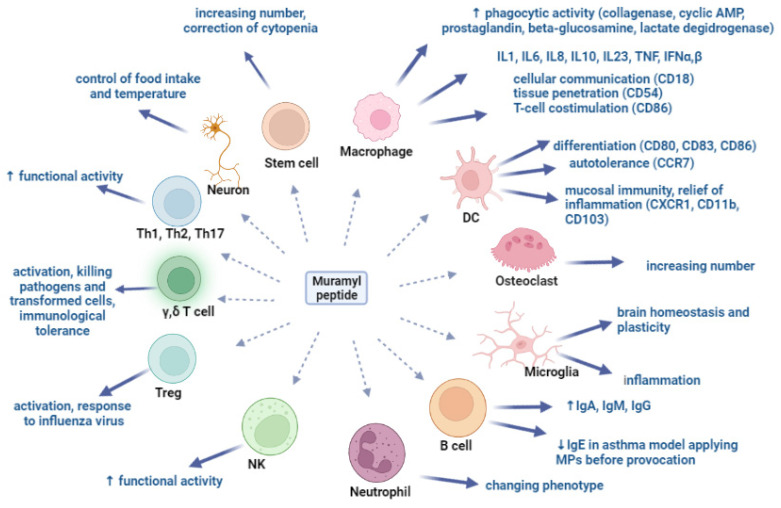
Muramyl peptides activation of various cell populations.

Muramyl peptides exhibit pronounced activity against mouse and human macrophages in vitro: their phagocytic and microbicidal ability increases due to an increase in the synthesis of cyclic adenosine monophosphate, superoxide radicals, collagenase, prostaglandins and an increase in the specific activity of beta-glucosaminidase, which is a component of the macrophage lysosome, and lactate dehydrogenase (Figure 2) [42,64]. Recorded changes were observed in peritoneal macrophages, in macrophages isolated from the bone marrow of animals, and also in macrophages isolated from the milk of healthy women [64].

Under the action of muramyl peptides, macrophages synthesize cytokines IL-1, IL-6, IL-8, IL-23, IFN α,β [65,66,67], and regulate the expression of membrane markers, including HLA-DR and adhesion molecules CD18, CD54, CD86 [67,68,69]. CD18-integrin is involved in the implementation of intercellular communication; CD54-Inter-Cellular Adhesion Molecule 1 (ICAM-1,) is required for tissue penetration; and CD86 is a membrane protein of the immunoglobulin family that acts as a co-stimulatory signal in antigen-dependent activation of T-lymphocytes. Thus, muramyl peptides stimulate macrophages to eliminate the pathogen, and to synthesize cytokines and mediators of innate immunity, as well as membrane-associated activation markers, providing the possibility of migration to the site of inflammation and interaction of immune system cells necessary for the implementation of adaptive immunity. Simultaneous stimulation of macrophages with MDP and TLR4 and TLR9 receptor agonists contributed to a significant increase in the synthesis of TNF, IL-1, IL-6, and IFN-α and IFN-β [45,70,71,72,73]. The synergy of the action of muramyl peptides with LPS and IFN on macrophages was the basis for the introduction of MPs as components of antibacterial and antiviral vaccines [74,75]. Interesting results were obtained when MPs were injected into the crushed sciatic nerve of rats [76]. It turned out that the intrafascicular injection of MDP activates macrophages that enter the damaged nerve and support the lengthening of the regenerating axon. The introduction of MDP accelerated the process of axon length increase by 15.5% after 5 days and by 18.3% after 3 weeks [76].

The role of muramyl peptides in the differentiation of dendritic cells (DCs) was determined. Muramyl dipeptide MDP-Lys (L18) increased the expression of CD80, CD83, CD86, and CD40, but not HLA-DR, and stimulated the production of tumor necrosis factor-alpha (TNFα), IL-6, IL-8, IL-10 and IL-12 (p40) human DCs. In addition, DCs treated with MDP-Lys showed an increased antigen-presenting function compared to untreated DCs [77]. Similar results were also obtained with GMDP [69]. Dendritic cells isolated from the blood of healthy donors who used the drug based on GMDP for 10 days changed their phenotype: the expression of differentiation markers CD80, CD83, and CCR7, which is responsible for DC recruitment to secondary lymphoid organs, increased. Constitutively expressed CCR7 provides autotolerance, as well as tolerance to food and inhalation antigens [78,79]. The expression levels of the XCR1, CD11b, and CD103 genes also increased. The XCR1 receptor plays a leading role in maintaining the homeostasis of mucosal immunity and the formation of memory CD8+T cells [80,81]. In addition, the expression of XCR1 on DC is required for the maturation of regulatory T cells, for the relief of inflammation, the maintenance of autotolerance, and for a cytotoxic immune response [82,83]. The discovered ability of GMDP to increase the expression of XCR1 and CD103 demonstrates the ability of GMDP to control excessive inflammation in mucosal tissues.

MDP induced osteoclast formation, showing synergy with LPS, and did not affect osteoclastogenesis in mice treated with the parathyroid hormone [84,85]. Macrophages of bone tissue osteoclasts resorb bone tissue and, together with osteoblasts, regulate bone remodeling.

Macrophages of the central nervous system (microglia) maintain homeostasis and brain plasticity. In an experimental model, activation of the NOD1 receptor by muramyl peptides enhanced the inflammatory response caused by microglia and exacerbated brain damage after intracerebral hemorrhage in mice [86].

An increase in antibody titer under the influence of MP, confirmed in numerous experimental and clinical studies, can serve as evidence of their activating effect on B-lymphocytes.

The first studies of muramyl peptides on lymphocytes were carried out using laboratory animals [3,87,88]. MDP at a concentration of 0.1 to 1 mg/kg provoked lymphocytosis, and at 10 mg/kg lymphocytopenia and an increase in the number of young stab neutrophils and monocytes. MPs by themselves did not induce antibody synthesis [89,90], but exhibited the properties of adjuvants when administered together with an antigen [75,88,91]. The adjuvant has no antigenic determinants and no antibodies are produced against it, but it can enhance the stimulatory response to compounds that have little effect on their own [92]. MPs exhibit adjuvant properties and potentiate the action of drugs and vaccines [4,93].

The ability of MPs to increase the titers of immunoglobulins was the basis for studying the mechanism of their action and introducing them as components of vaccines [3,87,89,94], since effective animal vaccines alum and Freund’s adjuvant have not been used in humans. MDP was the minimal structure of peptidoglycan included in Freund’s complete adjuvant. The study of numerous MDP derivatives showed that muramic acid and the presence of L-Ala and D-iGln are critical for the manifestation of biological activity [95]. Later, it was shown that the disaccharide-containing MP, GMDP, which is formed, in particular, under the action of lysozyme, increases the titer of antibodies to bovalbumin M much more efficiently than MDP [87]. The developed method for the synthesis of GMDP using a disaccharide obtained from bacteria and a compound with dipeptide turned out to be less expensive than previously proposed, which served as the basis for the development of a drug based on it. It should be noted that the adjuvant activity of MDP was largely expressed against ovalbumin and, to a much lesser extent, against sheep erythrocytes [96]. It is very important that muramyl peptides increase the immune response to low doses of antigen when other adjuvants (FCA, FIA) are not effective [97]. At the same time, MDP stimulated the production of immunoglobulins of the IgG1 subclass. MPs can also increase IgG2a titers [98].

The influence of MPs on the levels of IgA in the oral cavity of humans and animals [39,40,99], in the blood serum of humans [100] and animals [99,101], and in extracts of animal feces [101] was registered. It is interesting that 2-fold intranasal administration of MDP mice as an adjuvant with the joint administration of the recombinant urease protein (rUr) of Helicobacter pylori did not affect the increase in the titer of IgA and IgG in the blood serum; a composition of several adjuvants turned out to be effective [101]. Conversely, in extracts of animal feces, IgA was detected after the introduction of rUr with MDP and was absent in the composition of adjuvants [101].

MPs reduce the IgE titer in individuals with allergic diseases in the case of taking MPs at the stage of remission, as well as in animals with the experimental model of asthma with the preliminary administration of MPs before allergen sensitization, while the severity of the inflammatory process also decreased [40,98].

When mice were immunized with the Huntavirus vaccine, muramyl peptides B30-MDP and MDP-Lys(L18) showed an adjuvant effect and stimulated elevated levels of IgG1 and IgM, while no increase in IgG2 and IgG3 was observed [102]. Muramyl peptides increased antibody titers in mice immunized with a phage suspension [103].

Thus, MPs affect the synthesis of immunoglobulins. The intensity of the impact depends on many factors. The structure of MPs, their introduction into liposomes, route of administration, duration, dose, and properties of the antigen are obvious factors. However, it is additionally necessary to take into account other factors that can have a decisive effect, such as the duration of the early administration of the MPs to the antigen, in which not only the activating effect of the MPs is recorded, but also the suppressive one [40,104]. In addition, the adjuvant properties of MPs depend on the composition of the composition [105] and may depend on the presence of IL-2 and IL-4 [106].

Muramyl peptides change the phenotype and functional activity of neutrophil granulocytes [107,108,109] and natural killer cells (NK cells) [110,111], and activate Th17 [112] and regulatory T cells (Tregs) in response to influenza virus infection [113]. Depending on the microenvironment, MPs polarize the immune response towards Th1 or Th2 [114,115]. Muramyl peptides increase the functional activity of cytotoxic γδ T cells [52,116,117], which are involved not only in the destruction of pathogens and transformed cells, but also in the provision of immunological tolerance.

The effect of MPs on various immunocompetent cells is expressed in the induction of the synthesis of granulocyte colony-stimulating factor, which activates leukopoiesis and corrects cytopenias [118,119], and hematopoiesis-stimulating activity is also manifested in monotherapy [89,120].

Interesting data on a 1.9-fold increase in the number of multipotent bone marrow stem cells were obtained in bone marrow transplants of mice from bone marrow donors who received single injections of MDP 24 h before transplantation [121]. These results confirm previously obtained data on the correction of cytopenia during chemotherapy [119].

Modern studies have significantly expanded our understanding of the prevalence of muramylpeptide sensors. It turned out that muramylpeptides are able to cross the blood–brain barrier, directly activate brain neurons through NOD2 receptors, and change the activity of a subset of brain neurons that express Nod2 (Figure 2). Activation of Nod2 in the inhibitory neurons of the hypothalamus was found to be necessary for proper control of appetite and body temperature. It was noted that the interaction of MDP with NOD2 affects the feeding behavior of mice, significantly reducing their appetite. In the absence of the NOD2 receptor in neurons, these neurons no longer interact with muramyl peptides, and as a result, the brain loses control over food intake and body temperature. Mice gain weight and become more susceptible to developing type 2 diabetes, especially older females [122].

## 5. Muramyl Peptide Regulation of Intracellular Signaling Pathways

Muramyl peptides can appear inside cells during active transport with the help of the dipeptide transporter hPepT1, a pore-forming Pannexin-1, during pinocytosis with the capture of dissolved fragments of degraded peptidoglycan, during phagocytosis of bacteria, during lysosomal degradation or during death of bacteria that have penetrated into the cell [123,124,125], and bacteria can also introduce peptidoglycan through pathogenicity islands [126].

At the same time, the hPepT1 transporter delivers ligands only for the NOD2 receptor, but not for NOD1, into the cells [120]. The intracellular sensor of muramyl peptides is the NOD2 protein, which, after interacting with the MP, triggers a cascade of numerous downstream reactions, resulting in the elimination of the pathogen. It is believed that MPs are recognized by the LRR fragment of the NOD2 protein, the NACT domain is responsible for ATP activity and self-oligomerization, and two CARD domains are responsible for protein–protein interactions [127].

The repulsion of the pathogen attack seemed to be the main function of the NOD2 protein, with key amino acid residues that are evolutionarily conserved from bony fish to mammals [128]. Mutations in the NOD2 protein are associated with autoinflammatory diseases [129,130], Crohn’s disease [56], Blau’s syndrome and sarcoidosis [131,132,133].

The interaction of the MPs with the NOD2 protein changes the protein conformation and CARD domains are able to initiate the formation of multiprotein complexes, the first of which is RICK (receptor-interacting serine-threonine kinase, also known as RIP2 or RIPK2) [134]. RICK then acquires the ability to interact with LUBAC and TRAF3; in the first case, the nodosome multiprotein complex is assembled with activation of the transcription factor NF-kB, which induces the expression of pro-inflammatory cytokines and mediators; in the second case, interferon regulatory transcription factor 3 (IRF3) is activated and the IFNα and IFNβ genes are expressed [53]. LUBAC, the ubiquitin linear chain assembly complex (LUBAC), consisting of HOIL-1, SHARPIN, and the catalytically active HOIP subunit, is an E3 ubiquitin-ligating enzyme that, together with ubiquitin-activating (E1), ubiquitin-conjugating (E2) enzymes, performs post-translational protein modification ubiquitination. Protein chains ubiquitinated at the K63 lysine residue are recognized by the ubiquitin-binding domains TAB2 or TAB3 [135,136], which leads to the involvement of the TAK/TAB complex, as well as LUBAC [133]. LUBAC then ensures efficient recruitment of NEMO and hence the NEMO/IKKα/IKKβ (NEMO/IKK) complex. These two functional units then jointly trigger the activation of the NF-κB and MAPK signaling pathways [137].

Translocation of NF-kB to the nucleus is preceded by polyubiquitylation of IKKγ, an inhibitor of the NF-κB (IκB) kinase complex (IKK complex), which also consists of IKKα and IKKβ. This is followed by phosphorylation of IKKβ, and phosphorylation of IκB [138]. Activation of NF-κB is caused by the activation of mitogen-activated protein kinases (MAPKs), such as extracellular signal-regulated kinase (ERK) and amino-terminal JUN kinase (JNK) p38MAPK [139,140]. At the same time, NOD2-mediated activation of p38 and JNK was required for the production of pro-inflammatory cytokines, nitric oxide (NO), and iNOS expression in response to *Mycobacterium abscessus* [141]. Intranasal administration of MDP reduced bacterial replication in vivo and thus improved lung pathology in mice infected with *M. abscessus* [141].

It turned out that NOD2 can bind procaspase-1 through the interaction of their CARD–CARD domains to induce the secretion of interleukin-1β (IL-1β) [142]. Activated NOD2 (but not NOD1) also interacts with the intracellular molecule GRIM19 (gene associated with retinoid-IFN-induced mortality 19) [143]. This protein is located on the inner membrane of mitochondria and is homologous to subunit 13 of the 1-alpha NADH dehydrogenase subcomplex of the electron transport chain. The activity of GRIM19 is associated with mitochondrial respiration, and its deficiency leads to exacerbation of the activity of the mitochondrial respiratory chain [144]. GRIM19 attenuates the progression of obesity by controlling adipocyte differentiation, enhances glucose and lipid metabolism in the liver [145], induces p-53-dependent apoptosis in oncotransformed cells [146], increases the level of IFNγ, and regulates the balance of Th17/Treg cells [147]. The CARD domain of the NOD2 protein interacts with other proteins containing such domains and thus may be regulated by other members of the NLR family. In particular, CARD12, also known as IPAF, can bind to NOD2 and inhibit IL-1β production [142]. MDP, by activating NOD2, is a very potent inducer of matrix metallopeptidase 9 (MMP-9) [148]. MMP-9 is a protease involved in the degradation of extracellular matrix collagen and contributes to the pathogenesis of *Streptococcus pneumoniae.* MDP had dual effect on IL-1 synthesis. MDP binding to NOD2 induced the activation of Interleukin-1 receptor-associated kinase 1 (IRAK-1) and suppressed the activation of IRAK-1 on human mononuclear macrophages during long-term exposure. At the same time, the production of pro-inflammatory cytokines TNF-alpha, IL-8, and IL-1beta was significantly reduced [149]. Moreover, NOD2 triggers the Notch1 signaling response. The interaction between NOD2 and Notch1-PI3K complex promotes macrophage survival, reduces TNFα/IFNγ-induced apoptosis, and modulates the expression of IL-10 and a number of genes with anti-inflammatory functions [150,151].

**Figure 3:**
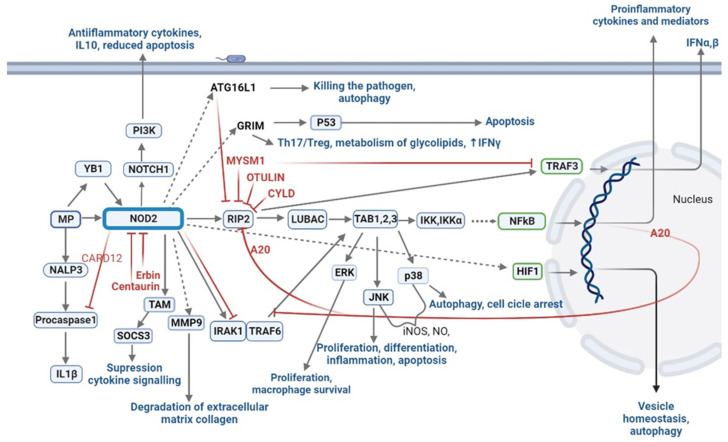
Positive and negative regulation of inflammation by muramyl peptides.

It is very important that when pro-inflammatory reactions are activated, the processes of negative regulation are triggered in order to stop the inflammatory response (Figure 3). A20 is a ubiquitin-modifying enzyme that limits signals induced by tumor necrosis factor receptor (TNF), Toll-like (TLR), and NOD2 receptors in vitro and in vivo [152]. A20 performs these functions both by deubiquitinating K63-associated polyubiquitin chains and by adding K48-associated polyubiquitin chains to RIPK2 and TRAF6 signaling proteins [152]. Similarly to A20, proteins OTULIN, CYLD, and MYSM1, they downregulate RIPK2 by deubiquitinating [153,154,155]. RIP2 tyrosine kinase activity has been found to play a dual role in NOD2-dependent autophagy: RIP2 sends a positive autophagy signal via p38 MAPK activation and attenuates autophagy repression mediated by PP2A phosphatase [151,156]. Another example of negative regulation is NOD2-dependent activation of the SOCS-3 protein, which facilitates the dissociation of NOD2 and its chaperone Hsp90, accelerating ubiquitination and proteasomal degradation of NOD2 [157]. The proteins Erbin, Centaurin β1, and ATG16L1 directly interact with NOD2 and prevent its biological activity [158,159,160]. Regardless of the adapter RIP2 and the transcription factor NF-kappaB, Nod1 and Nod2 recruit the autophagy-related protein 16 like-1 (ATG16L1) autophagy protein to the plasma membrane at the site of bacterial entry, inducing autophagy and killing the pathogen [161]. ATG16L1 negatively regulates NOD-induced inflammatory responses by interfering with RIPK2 adapter polyubiquitination and preventing nodosome formation [53,124].

Examples of the downregulation of inflammation provide new insight into how these signals are controlled and physiologically limited. Understanding the complexity of coordinating numerous stages of molecular interactions explains the positive effect of the use of drugs based on muramyl peptides, and reveals new possibilities for effective therapy and prevention [4,162].

NOD2 protein is not the only intracellular MPs sensor. The YB1 protein was shown to interact directly with GMDP and activate NOD2 [163]. Cryopyrin, a member of the NLR family, also known as NALP3, is also activated by MDP independently of NOD2 [164]. However, in this case, the molecule activates procaspase-1 and therefore promotes the secretion of IL-1β. On the other hand, it has been shown that ligands other than muramyl peptides exist for the activation of the NOD2 protein, for example, viral RNAs [53], sulfatides (3-O-sulfo-galactoceramide, 3-OSGC), and sulfolipids (*Mycobacterium* sp. specific sulfolipid-1) [165]. Autophagy and NF-kB signaling are separate functions of NOD2 and they also occur in cells independent of bacterial infection. It is very important that 3-OSGC is an endogenous NOD2 ligand, regulating NOD2 function in vesicular homeostasis, but this does not affect Nf-kB signaling. At the same time, the method of penetration of 3-OSGC located on the outer side of the cell membrane into the cytosol remains unclear; various flippases are considered as candidate transporters, the level of which is increased in Crohn’s disease [166]. A study by Nabatov et al. demonstrates that NOD2 is regulated by hypoxia-inducible transcription factor-1 (HIF-1), participates in autophagy, maintaining the acidity of intracellular vesicles [165].

The Tyro3, Axl, and Mer (TAM) receptors inhibit the signaling outcomes of TLR3, TLR4, and TLR9. TAM-deficient mice develop lymphoproliferative diseases and various autoimmune diseases including arthritis, pemphigus vulgaris and lupus, Mer−/− mice show increased TNF-dependent mortality upon acute injection of LPS. Therefore, TAM receptors are critical for the downregulation of pro-inflammatory cytokines under the conditions of chronic NOD2 stimulation seen in the intestinal environment [167].

Interest in the mechanisms of regulation of immunity in normal and pathological conditions is steadily increasing. The coordination of many multidirectional processes is crucial for maintaining homeostasis. To accurately record the results of studies and visualization, systems biology approaches are used based on the compilation of cause-and-effect relationships, taking into account the type of cells, the registration of molecules at the level of transcriptome, proteome, and metabolome [168,169,170]. Machine-readable languages and various ways of building networks of mutual influences have been formed, which serve as the basis for drug design and the development of effective methods of therapy and prevention.

## 6. Conclusions

Low molecular weight fragments of bacterial peptidoglycan affect the representatives of the microbiological community inhabiting the skin and mucous membranes, and affect their life cycle and antagonistic relationships.

Muramyl peptides affect not only the immunocompetent cells of a macroorganism, but also almost all cells and tissues, participating in the activation and differentiation of cells, changing their phenotype, promoting migration and stimulating hematopoiesis.

Muramyl peptides of bacterial origin are bioregulators of intracellular processes. They signal the presence of bacteria and trigger not only the pro-inflammatory processes necessary to eliminate the infection, but also the anti-inflammatory processes necessary to stop inflammatory processes and maintain tolerance to the microflora.

A broad spectrum of microorganism-derived low-molecular-weight peptidoglycan fragments regulate host homeostasis and trigger and down-regulate immune responses that, depending on the context, may have opposite effects.

## Data Availability

Not applicable.
